# Interplay between Safety Climate and Emotional Exhaustion: Effects on First Responders’ Safety Behavior and Wellbeing Over Time

**DOI:** 10.1007/s10869-022-09869-1

**Published:** 2022-12-22

**Authors:** Jin Lee, Christian J. Resick, Joseph A. Allen, Andrea L. Davis, Jennifer A. Taylor

**Affiliations:** 1 Department of Psychological Sciences, Kansas State University, Manhattan, USA; 2 Department of Management, LeBow College of Business at Drexel University, Philadelphia, USA; 3 Department of Family & Preventive Medicine, University of Utah Health, Salt Lake City, USA; 4 Department of Environmental & Occupational Health, Dornsife School of Public Health at Drexel University, Philadelphia, USA

**Keywords:** EMS first responders, Safety climate, Emotional exhaustion, Safety behavior, Wellbeing

## Abstract

Various job demands continuously threaten Emergency Medical Service (EMS) first responders’ safety and wellbeing. Drawing on Job Demands–Resources Theory, the present study examines the effects of the organizational context—safety climate—and the psychological context—emotional exhaustion—on safety behaviors and wellbeing over time. We tested our hypotheses in a longitudinal study of 208 EMS first responders nested within 45 stations from three fire departments in US metropolitan areas over 6 months during the beginning of the COVID-19 pandemic. Multilevel modeling showed that the relationship between safety climate and safety compliance behaviors can be attenuated when EMS first responders experience high emotional exhaustion. Emotional exhaustion was also negatively associated with morale while safety climate was positively associated with morale. Additionally, EMS first responders experienced increased depression when their emotional exhaustion levels were high. Higher safety climate was associated with decreased depression when emotional exhaustion was within a low-to-medium range. Higher safety climate was also associated with lower absolute levels of depression across the entire range of emotional exhaustion. These findings suggest that promoting safety climate and mitigating emotional exhaustion can augment EMS first responders’ safety behaviors and wellbeing.]

Emergency medical service (EMS) first responders provide field-based medical response that serves as a critical public health safety net. They are commonly exposed to biohazardous materials, respond to calls in high-risk and uncertain environments, and treat patients along the spectrum from general assistance to life-or-death situations (Donnelly, 2012). The physical and psychological demands of the work are considerable and the consequences of these demands can be dire, such as the loss of life. In fact, high rates and frequencies of safety incidents, diseases, accidents, and injuries (Kahn, 2006; Louzado-Feliciano et al., 2020; Maguire et al., 2005; Valdez et al., 2015) have been reported among first responders. Additionally, a high prevalence of depression and anxiety problems (Bergen-Cico et al., 2015), PTSD (Feldman et al., 2021), and substance abuse (Davis et al., 2014), and suicide (Vigil et al., 2019) have been noted. According to Jones (2017), the mental health profile of first responders is characterized by PTSD, depression, suicidality, anxiety, alcohol use, and sleep disturbances.

To prevent the collapse of this public health safety net, it is imperative to examine the salient conditions of first responders’ physical and psychological challenges. In this study, we focus on two contextual aspects—the work context of the fire station and the psychological context of the individual—critical to understanding how EMS first responders address these physical and psychological challenges. Regarding the organizational context, safety climate is an important organizational resource that encourages members to engage in actions that protect their safety and health (Griffin & Curcuruto, 2016). This resource is especially important in high demand contexts such as EMS work (Demerouti et al., 2001; Schaufeli et al., 2009). The psychological context involves the emotional, affective, and personality attributes of the individual that impact one’s capacity to learn and respond to emerging events (Burnett et al., 2009; Moras et al., 1993; Radomski, 2007; Schuster & Nykolyn, 2010). Emotional exhaustion is a central component of burnout that reflects depleted psychological resources that impair cognitive schemes, judgments, and behavior (Alarcon, 2011; Taris et al., 2005). Because prior theory and research on burnout recognized emotional exhaustion as the most essential and representative attribute of burnout (Te Brake et al., 2008; Wright & Cropanzano, 1998), we focus specifically on emotional exhaustion to capture the individual psychological context in the current study. If first responders are emotionally exhausted, they are likely to feel overextended, fatigued, and distracted leading to compromised situation awareness and maladaptive behavior (Mathisen & Bergh, 2016; Sneddon et al., 2013; Wright & Cropanzano, 1998). As both the organizational and psychological contexts co-exist, we further argue that the characteristics of each are likely to facilitate or inhibit the effects of the other.

According to Job Demands–Resources theory (J-DR; Demerouti & Bakker, 2011; Demerouti et al., 2001), job demands and resources are interdependent. High resources can bolster worker motivation while high demands can dampen it. High demands can exacerbate strain while adequate resources can alleviate it. Safety climate can be viewed as a specific organizational resource, while emotional exhaustion can be viewed as a specific psychological demand. Although emotional exhaustion has been predominantly attributed to excessive and repeated job strain (Bakker and Demerouti, 2007; Bakker & De Vries, 2021), studies indicate that emotional exhaustion may affect how individuals respond to job demands and resources. Specifically, Qian et al. (2020) contended that emotional exhaustion depletes personal resources. Supporting this view, emotional exhaustion has been linked to impairment of self-efficacy, emotional intelligence, and social support (Molero Jurado et al., 2018). Also, it was shown that emotional exhaustion can aggravate wellbeing at work, coping resources, work ability, and engagement (Lee et al., 2019a, 2019b, 2019c; Voltmer et al., 2018). Moreover, a reciprocal relationship between emotional exhaustion and job demands has been noted (Ângelo & Chambel, 2015; Shahidi et al., 2022; Tone Innstrand et al., 2008). Therefore, we view that emotional exhaustion is not just an end-outcome of extended exposure to stress. It also functions as a specific form of secondary or derivative psychological demand that requires effort and drains energy. In turn, the positive relationship between safety climate and safety behavior is likely to be weakened if workers’ motivation to comply with safety behaviors is compromised due to high psychological demands from emotional exhaustion. On the other hand, the potential negative effect of emotional exhaustion can be mitigated if workers perceive that their organization offers adequate resources. An organization with a positive safety climate will offer resources such as protection and support for members’ health and wellbeing in general (Leitão et al., 2021; Taylor et al., 2019).

The current study aims to make three unique contributions to the safety climate and wellbeing literatures. First, an emerging stream of research indicates that safety climate has implications for both employee safety and wellbeing (Huang et al., 2016; Taylor et al., 2019). To date, however, safety climate research has largely neglected the psychological context of individual employees in examining the effects of safety climate for individuals’ occupational safety behaviors and wellbeing across the work and personal domains. This is an important oversight because psychological demands from work drain employee resources and impair both cognitive and motor functioning, further compromising performance and adaptivity. These effects are likely to be particularly apparent among employees working in high demand occupational contexts such as health care and the fire service.

Second, our study extends Maslach and Leiter’s (2016) burnout model by demonstrating that the organizational context, in the form of safety climate, has implications for understanding the effects of employee emotional exhaustion on behavior and wellbeing. Specifically, the present study sheds light on how employees whose psychological context is impaired are able to respond and adapt to the evolving conditions in demanding occupational contexts.

Third, the present study embraces the view that safety climate and emotional exhaustion are dynamic, and that their interplay needs to be examined over time. To this end, the present study adopted a longitudinal study design to examine the unfolding effects of emotional exhaustion and safety climate on EMS first responders’ safety behavior and wellbeing across 6 months of the COVID-19 pandemic. There have been attempts to scrutinize emotional exhaustion (e.g., Burisch, 2002; Van Dierendonck et al., 2001) and wellbeing (e.g., Galais & Moser, 2009; Möhring et al., 2020) based on repeated measures designs. However, these studies used prospective designs with only two or three time points that focused on the lagged effects or used difference scores across time points. Moreover, our study was conducted in the early months of the COVID-19 pandemic. In crisis situations such as this, events evolve rapidly and unpredictably threatening organizational members’ wellbeing (Billings et al., 1980; James & Wooten, 2005). These conditions heighten the salience of contextual factors such as safety climate and emotional exhaustion within this high demand occupation. Figure 1 presents the study’s conceptual model, and Fig. 2 summarizes the hypothesized relationships graphically.

**Fig. 1:**
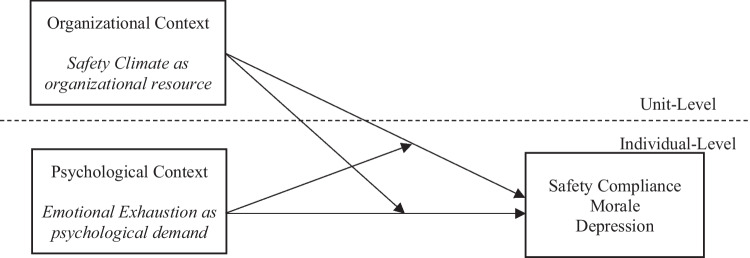
Conceptual model based on job demands–resources theory

**Fig. 2:**
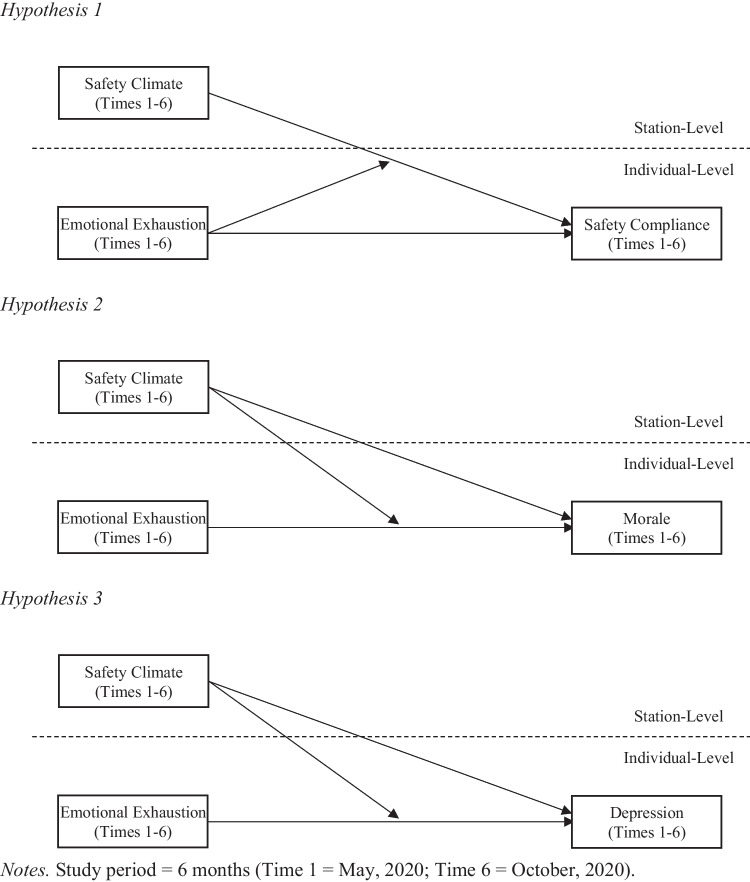
Hypothetical model of relationships*.* Study period = 6 months (time 1 = May, 2020; time 6 = October, 2020)

## Organizational Context of First Responder Safety: Safety Climate

Safety climate refers to workgroup members’ shared perceptions regarding safety policies, procedures, and practices (Zohar, 1980). Safety climate manifests at multiple levels within organizations. At the organization-level, safety climate encompasses shared perceptions of decision-makers’ prioritization of safety throughout the organization. In the fire service, this level is called the “department.” At the group-level, safety climate encompasses shared perceptions of supervisory practices that directly support safety protocols within the group (Zohar & Luria, 2005). In the fire service, this level is called the “station.”

Safety climate provides clear norms for safety and health among organization members and establishes congruent organizational policies and procedures that facilitate workplace safety and health (Clarke, 2010, 2012). Organizations with high safety climate are attentive and responsive to members’ concerns and needs for occupational safety and health. Also, they provide timely and practical support such as proactive risk management, compassionate and constructive feedback, and customized training programs. For EMS first responders, safety climate is an important organizational resource that motivates them to perform responsibly for their own safety and health through a series of interactional processes of sense-making and empowering (Griffin & Curcuruto, 2016). The messages, actions, and decisions of supervisory leaders signal the importance of safety as a group priority, thereby shaping members’ shared perceptions of the extent to which the environment is supportive of safety (Nahrgang et al., 2011; Zohar & Luria, 2004). Taylor et al. (2015) identified five major domains of occupational safety and health concerns for EMS first responders: assaults by patients, risks from motor vehicles, inadequacy of personal protective equipment use, relationships between emergency responders (e.g., teamwork, communication), and policies, procedures, and practices. These occupational hazards heighten the importance of safety climate for EMS first responders because there is a high risk of injury, illness, and poor wellbeing (Reichard & Jackson, 2010; Taylor et al., 2019).

## Psychological Context of Wellbeing of First Responders: Burnout–Emotional Exhaustion

Prolonged exposure to work environments that are physically and psychologically demanding can result in burnout (Halbesleben, 2006; Halbesleben et al., 2014; Shirom, 1989). Burnout is a state of physical and mental exhaustion due to chronic job stress. The present study focuses on a specific aspect of burnout, emotional exhaustion that concerns feelings of being psychologically overextended and depleted of psychological resources. Emotional exhaustion is the most representative and core symptom of burnout (Qian et al., 2020; Shirom, 1989; Wright & Cropanzano, 1998). It can lead to two other aspects of burnout involving depersonalization—disconnected feelings and cynical attitudes—and reduced personal accomplishment—a diminished sense of competence and compromised performance (Alarcon, 2011; Maslach & Jackson, 1981; Maslach et al., 1997; Taris et al., 2005; Vercambre et al., 2009). Lee and Ashforth’s (1996) meta-analysis showed that both job demands and resources were more strongly related to emotional exhaustion than either depersonalization or personal accomplishment. Research has noted that emotional exhaustion can negatively affect employee health, productivity, and wellbeing (Alarcon, 2011; Maslach & Leiter, 2016; Taris et al., 2005).

EMS first responders do frequent shiftwork and commonly undertake physically demanding tasks that entail heavy lifting, forceful exertions, and excessive reaching. They work under great time pressure in many circumstances while also addressing life-threatening situations. The emotional toll of providing care such as compassion fatigue and vicarious trauma can be considerable (Renkiewicz & Hubble, 2021). These compounding job demands make emotional exhaustion very likely among EMS first responders (Crowe et al., 2018, 2020). A greater concern is that emotional exhaustion can further impair their wellbeing. The present study concentrates on the after-effect of emotional exhaustion on two indicators of wellbeing, namely, morale and depression.

Any situation perceived to threaten one’s psychophysical resources can lead to behaviors to augment, restore, or prevent further loss of these resources (Hobfoll, 1989, 2011). When resources are draining without proper replenishment, ongoing stress can result in emotional exhaustion (Halbesleben, 2006; Halbesleben et al., 2014). The burnout model of Maslach and Leiter (2016) indicates that burnout and its key attribute of emotional exhaustion is not merely the ultimate outcome of stress, but is very likely to have further aggravating effects on health, productivity, and wellbeing. Thus, emotional exhaustion can be viewed as a psychological context that sends a warning signal to a person concerning their depleted resources and lack of ability to cope with stressful situations.

Specifically, if a worker experiences heightened levels of emotional exhaustion due to challenging work conditions (Van den Broeck et al., 2008; Xanthopoulou et al., 2007), this person might preserve psychophysical resources by engaging in less risk taking and more withdrawal behaviors at work. This process bears the resemblance to the flight response to stress which includes dismissive attitudes as well as avoidance and escape behaviors (Lindhardt et al., 2008; McCarty, 2016). Also, these processes suggest a potential for emotional exhaustion to negatively influence morale, which encompasses high confidence, proactive attitudes, and elevated job satisfaction (McKnight et al., 2001; Mishra et al., 1998). Studies have shown that high emotional exhaustion can be associated with low morale (Koeske & Kelly, 1995; Robinson-Kurpius & Keim, 1994; Roloff & Brown, 2011). Morale is closely associated with important organizational outcomes such as performance and productivity (Neely, 1999) and turnover intention (Verma & Kesari, 2020). Thus, proper management of emotional exhaustion is essential for promoting high morale in the work environment.

Moreover, emotional exhaustion experienced in the work domain can spillover, inducing depression in the personal domain over time (Koutsimani et al., 2019; Maslach & Leiter, 2016; Toker & Biron, 2012). Affective deterioration over the course of work can render individuals more vulnerable to psychological deterioration in a more global domain (Hobfoll, 2011; Huibers et al., 2007). The continued threat or loss of resources and inability to restore lost resources through coping mechanisms may trigger symptoms like diminished pleasure, persistent feeling of negative emotions, loss of interest, and disrupted daily functioning (Armon et al., 2008; Idris et al., 2014; Toker & Biron, 2012). These symptoms reflect depressive mood problems.

Accumulating evidence indicates that burnout (including its emotional exhaustion dimension) and depression are related, but distinct experiences of psychological distress. For example, meta-analyses estimate the correlation between burnout and depression between 0.49 (Meier & Kim, 2022) and 0.52 (Koutsimani et al., 2019) providing evidence that the constructs are not redundant but distinct across studies. Meier and Kim (2022) further demonstrated that burnout requires recurrent and negative experiences at work to develop to the extent that burnout overlaps with depression symptoms.

High levels of emotional exhaustion (Crowe et al., 2018) and depression (Kim et al., 2019) are common among first responders. Caldas et al. (2021) showed that first responders who were intensely involved in treating patients in demanding situations (e.g., COVID-19 pandemic) experienced heightened levels of emotional exhaustion and depression. The impact of depression can be grave, such as heart disease and compromised immunity (Kiecolt-Glaser & Glaser, 2002) and increased risk of suicide (Jones, 2017). Considering the potential impact of emotional exhaustion on depression and the dire outcomes of depression, the prevention and mitigation of depression through the proper management of emotional exhaustion is critical.

## Job Demands–Resources (JD-R) Theory and Hypotheses

The job demands placed on EMS first responders are a continuous threat to their personal safety and wellbeing (Taylor et al., 2016). Meanwhile, numerous forms of resources for EMS first responders such as psychological capital and organizational support for safety and wellbeing provide counter measures that facilitate safety and wellbeing. JD-R theory supports the contrary roles of job demands and resources. The present study also notes the potential interactions between job demands and resources (Demerouti & Bakker, 2011; Demerouti et al., 2001).

The JD-R theory suggests the relationship between job resources and motivation is moderated by job demands. However, there are opposing views concerning the direction of the moderation. According to the coping hypothesis (Bakker et al., 2010; Hakanen et al., 2005), the relationship between job resources and outcomes such as motivation and engagement can be amplified when job demands are high. In a work context with high job demands, the importance of job resources becomes more apparent. The coping hypothesis argues that individuals are more likely to utilize resources to reduce stress and cope with challenges in demanding circumstances. Alternatively, Bakker and Costa (2014) posited that the relationship between adequate job resources and positive outcomes can be weakened when job demands are high. The present study adopts this view because emotional exhaustion intensifies an employee’s susceptibility to reduced motivation and subsequent compromised adherence to stringent work protocols. Therefore, the positive effects of safety climate on safety compliance behaviors can be dampened when individuals experience high emotional exhaustion. In a similar vein, Bakker and Costa (2014) argued that burnout—especially emotional exhaustion—can undermine the gain cycle of daily job resources and job crafting. Furthermore, emotional exhaustion can weaken the positive relationship between work values and organizational citizenship behaviors (Liang, 2012) as well as the positive relationship between the quality of work life and its contribution to productivity (Leitão et al., 2021). Therefore, we propose that emotional exhaustion will function as a psychological demand and compromise the positive effect of station safety climate on safety compliance behaviors at the individual-level over time. Thus, we hypothesize the following:

- ***Hypothesis 1:*** *Over time, emotional exhaustion at the individual-level will moderate the positive relationship between safety climate at the station-level and safety compliance behaviors at the individual-level, such that the relationship will be weakened when emotional exhaustion is high.*
  The JD-R theory also proposes the buffer hypothesis, which suggests that the relationship between job demands and strain is moderated by job resources that can lessen unfavorable outcomes of job demands on job strain (Bakker et al., 2003, 2005; Xanthopoulou et al., 2007). Kahn and Byosiere (1992) contended that job resources can address the organizational properties that trigger specific stressors, alter the employees’ perceptions regarding the stressful and demanding situations, or curtail detrimental consequences of the situations. In this way, any stress–strain relationship can be alleviated by job resources.
  Supervisory implementation of organizational safety protocols, plus day-to-day guidance, recognition, and support for worker safety are instrumental in advancing station safety climate and promoting worker safety and wellbeing (Christian et al., 2009; Zohar & Luria, 2004; Zohar et al., 2014). In addition, the ongoing encouragement and reinforcement of safety protocols demonstrate a genuine sense of care and respect for the safety, health, and wellbeing of employees. These actions build trust between leaders and members, creating an environment in which EMS first responder’s sense of self-worth and self-efficacy can grow despite high job demands and emotional exhaustion. Thus, we propose that emotional exhaustion will have a detrimental effect on EMS first responders’ morale over time and that a favorable station safety climate will reduce the impact. We hypothesize the following:
- ***Hypothesis 2:*** *Over time, safety climate at the station-level will moderate the negative relationship between emotional exhaustion at the individual-level and morale at the individual-level, such that the relationship will be weakened when station safety climate is high.*
  Additionally, we propose that safety climate can signal the importance of employees’ comprehensive wellness. This encompasses both physical and psychological attributes of health, and prevents the spillover of work-related emotional exhaustion to a global or personal emotional domain. In fact, social and relational aspects of supervisory support, which are integral attributes of safety climate, were shown to be associated with increased job control, job satisfaction, and reduced stress (e.g., Hall, 2007; Huang et al., 2016; Kang & Kang, 2016). Along with enhanced job control, one can attempt to alter the work environment to better cope with emotional exhaustion. Greater job satisfaction and reduced stress can effectively mitigate the after-effect of emotional exhaustion. Also, past research has noted that supervisors’ emotional, informational, and instrumental support can augment confidence and self-efficacy while promoting positive mood among employees (e.g., Kadirvelu et al., 2012; Leahy-Warren et al., 2012). The station’s safety climate provides an organizational resource supporting safety initiatives and counterbalancing the demands of work environment. Subsequently, this creates space for individual first responders to take care of their own psychological welfare even when job demands are high and emotional exhaustion is likely. Therefore, we propose that station safety climate will function as an organizational resource that prevents emotional exhaustion from evolving into depression over time and hypothesize the following:
- ***Hypothesis 3:*** *Over time, safety climate at the station-level will moderate the positive relationship between emotional exhaustion at the individual-level and depression at the individual-level, such that the relationship will be weakened when station safety climate is high.*

## Method

### Sample and Procedure

Data were collected from fire department-based EMS first responders in three large metropolitan areas (population served *mean* = 1,328,500, *SD* = 12,020) located in different geographic regions of the USA including the West Coast, Southwest, and Northeast regions. Each department responds to a high volume of EMS calls annually (*mean* = 307,031, *SD* = 11,480) with calls split 80% (EMS) to 20% (fire) annually on average. For this study, during the peak of the first wave of the pandemic in April of 2020, we partnered with department and union leadership to recruit a random sample of EMS first responders, inviting them to complete six monthly surveys beginning in May ending in October. The study was approved by [name redacted for peer review] human subject’s institutional review board.

Under normal circumstances, the job demands on EMS first responders are a continuous threat to their personal safety and psychological wellbeing (Taylor et al., 2016), and high levels of emotional exhaustion (Crowe et al., 2018) and depression (Kim et al., 2019) are common within the profession. These effects may be compounded during the COVID-19 pandemic (Fu et al., 2021; Hoffman, 2020). Specifically, job demands placed on first responders were intensified because of increased safety precautions and expanded use of personal protective equipment (PPE) (Coto et al., 2020; Ventura et al., 2020). Moreover, early evidence indicates that medical first responders who were intensely involved in treating COVID-19 patients experienced heightened levels of emotional exhaustion and depression (Caldas et al., 2021).

With the assistance of each department’s labor union, safety officers, and chief officers responsible for EMS, we recruited 200 or more EMS first responders from each department. Potential participants were asked to commit to completing a survey once a month for 6 months beginning in May 2020 with a 1-month lag between survey requests. A total of 800 EMS first responders initially enrolled in the study with response rates as follows: time 1 = 478 (59.8%), time 2 = 364 (45.5%), time 3 = 292 (36.5%), time 4 = 444 (55.5%), time 5 = 347 (43.4%), and time 6 = 255 (31.9%). Each month a fire department champion sent an email invitation directly to each participant that contained a link to the monthly survey.

Data from self-report measures such as those used in the current study are susceptible to bias due to each individual respondent’s unique response pattern. The transient states of respondents (e.g., response styles, moods) might contribute to common method variance bias (Podsakoff & Organ, 1986; Steenkamp & Baumgartner, 1998). We took the following steps to minimize bias from common method variance. First, we collected data over multiple periods in accordance with the key recommendation to address the threat of common method bias (Ostroff et al., 2002; Podsakoff et al., 2003). In a longitudinal survey design, temporal separation breaks up the influence of respondents’ transient response styles and moods, minimizing the potential for common method bias, which leads to artificially inflated correlations between a predictor and its outcomes (Rindfleisch et al., 2008). Second, respondents were anonymous, and the distinctiveness of the measures was examined via a series of confirmatory factor analyses in line with the suggestions by Conway and Lance (2010).

We retained data from participants who completed three or more surveys because a minimum three waves of data are required for the detection of longitudinal trends in terms of stability and change (Everaert & Joormann, 2020; Singer & Willett, 2003). At the same time, we retained data from fire stations with three or more respondents to ensure the reliability and representativeness of the station-level safety climate (supervisor support for safety) scores. The final sample included 208 EMS first responders nested within 45 stations across the 3 departments. The average number of respondents from each station was 4.62 (SD = 2.15). Among the respondents, 37 (17.8%) completed 6 surveys, 51 (24.5%) completed 5 surveys, 49 (23.6%) completed 4 surveys, and 71 (34.1%) completed 3 surveys. The incomplete nature of the data resulted in an overall missing data rate of 29.0% across the entire set of study measures across the six time points. The pattern of missingness in our data was missing completely at random (Little, 1988; Tierney et al., 2021) with *χ*^2^ = 830.08 (*df* = 795, *p* = 0.19) and not systematically associated with departmental membership, demographic variables such as age, sex, or tenure, as well as other study variables.

The missing variables within the missed survey administrations were handled at the composite score-level with the predictive mean matching (PMM) multiple imputation method (Little, 1988; Rubin & Schenker, 1986). The procedure generates realistic data values preserving the original data distribution and interrelationships among variables by randomly borrowing an observed value from existing data points (donors) that have a similar mean using multiple regression. The values are robust even when sample size is less than 100 (Kleinke, 2018) and with non-normal data with 30% of missingness or less (Kleinke, 2017). A recent simulation study showed that PMM yielded the least bias compared to the list-wise deletion and Poisson imputation methods when the missingness was 30% (Bengtsson & Lindblad, 2021). Moreover, studies have consistently demonstrated that PMM-based imputation with equal or greater than 100 data point donors are associated with negligible increase in bias and estimation error as the number of missing cases increases up to 40%, regardless of the specific method of donor selection strategy (Kleinke, 2018; Schenker & Taylor, 1996). In our study, the numbers of intact donors for each study variable ranged from 113 to 171 with a mean of 147.7 (SD = 18.4). For this study, PMM was conducted using the multivariate imputation by chained equation (MICE) method which can handle diverse types of variables and data complexities due to bounds or survey skip patterns (Horton & Lipsitz, 2001; Van Buuren & Groothuis-Oudshoorn, 2011).

Participants in the final sample were predominately male (87.50%) with a mean age of 36.62 years (*SD* = 6.72),and an average of 9.2 years of service in the fire department. Participants represented a range of ethnicities: 62.50% white/non-Hispanic, 15.38% Hispanic, 7.69% Black, and 14.42% other.

### Measures

#### Safety Climate

We assessed station safety climate using the 7-item *Supervisor Support for Safety* dimension of the *Fire Service Organizational Culture of Safety* (FOCUS) survey (Taylor et al., 2019) which was designed to assess group-level safety climate. The FOCUS survey builds on Zohar and Luria’s (2004) multilevel conceptualization and offers a fire service specific measure of safety climate that enables researchers and practitioners to assess safety climate at the organization (i.e., fire department) and group (i.e., fire station) levels. This measure has demonstrated satisfactory psychometric properties and construct validity in prior research (see Taylor et al., 2019). Participants responded using a 5-point Likert-type scale (1 = *strongly disagree* to 5 = *strongly agree*). Sample items for this and all measures are included in the Appendix. Acceptable internal consistency reliabilities were found at each time point with a mean α of 0.92 across the six time points (range = 0.90–0.93). Safety climate in terms of supervisor support for safety is a shared construct conceptualized and operationalized at the station-level. Therefore, we aggregated the individual-level responses to the station-level. Across the six time points, we found acceptable levels of within station agreement based on index *r*_wg(j)_ with a uniform null distribution (mean = 0.96; range = 0.95–0.96) and a slightly skewed null distribution (mean = 93; range = 0.91–0.94).

#### Emotional Exhaustion

We measured emotional exhaustion using the 5-item measure developed and validated by Maslach and Jackson (1981). Participants were asked to reflect on the past month and respond using a 5-point Likert-type response scale (1 = *strongly disagree* to 5 = *strongly agree*). The mean Cronbach’s alpha = 0.94 (range = 0.92–0.95).

#### Safety Compliance

Safety compliance is a form of safety behavior that refers to the degree to which members act in accordance with established safety protocols, processes, and standards. Safety compliance behaviors are the core safety activities of formal work procedures that are critical for a minimum level of workplace safety (Griffin & Curcuruto, 2016). Safety compliance behaviors pertinent to EMS first responders were assessed with 7-items developed for this study. The items on the safety compliance behavior measure (see Appendix) correspond to specific behavioral indicators of safety compliance. Participants were asked to reflect on the past month and respond using a 5-point Likert-type scale (1 = *strongly disagree* to 5 = *strongly agree*). The mean Cronbach’s alpha = 0.73 (range = 0.62–0.83).^1^ Because this was a newly created measure, we conducted a series of confirmatory factor analyses specifying independent error terms. A single-factor model yielded satisfactory fit (Hu & Bentler, 1999) across all six time points (mean *χ*^2^ = 120.85 (range = 88.88–174.23, *df* = 14), mean CFI = 0.998 (range = 0.997– 0.999), and mean SRMR = 0.000 (range = 0.000–0.000)).

#### Morale

We assessed morale using a four-item scale targeted at healthcare workers designed by Sexton et al. (2006). Participants were asked to reflect on the past month and respond using a 4-point scale (1 = *not at all*, 2 = *several days*, 3 = *more than half days*, 4 = *nearly every day*). The mean Cronbach’s alpha = 0.82 (range = 0.80–0.84).

#### Depression

We assessed depression using a two-item scale developed and validated by Kroenke et al. (2007), which is one of the most widely used scales to gauge the key affective symptoms of depression. Reliability, validity, and clinical utility of this depression measure have been extensively supported by empirical studies (Löwe et al., 2005; Richardson, et al., 2010; Yu et al., 2011), and the findings were confirmed by a recent meta-analysis by Manea et al. (2016). Participants were asked to reflect on the past month and respond using a 4-point scale (1 = *not at all*, 2 = *several days*, 3 = *more than half days*, 4 = *nearly every day*). The mean Cronbach’s alpha = 0.87 (range = 0.79–0.94).

#### Controls

We controlled for gender and tenure at the individual-level. Prior studies have shown a relationship between gender and depression (Nolen-Hoeksema & Hilt, 2009; Piccinelli & Wilkinson, 2000) and tenure has been positively associated with psychological strain in healthcare professionals (Kelly et al., 2015). Following Becker et al. (2016), we tested the hypotheses with and without controls to determine the robustness of the effects.

### Analytical Strategy

We tested the hypotheses using multilevel modeling for repeated measures conducted with the “lme4” package for R (Bates et al., 2015). Unlike a latent growth curve modeling approach which focuses primarily on the general change pattern of repeated measures (e.g., growth or decline), a multilevel modeling approach enables the examination of how time-varying (and/or time-invariant) independent variables are associated with a time-varying dependent variable after controlling for the time effect. Moreover, flexible specification of the interactions with time is available in the multilevel modeling approach. In the latent growth curve modeling framework, time is not specified as a variable and the meaning of interactions with time is less clear (Li et al., 2000; McNeish & Matta, 2018). Statistical significance of the coefficients was examined with “lmerTest” package for R (Kuznetsova et al., 2017) because the “lme4” package does not provide degrees of freedom and *p*-values. We specified 4-level models with department specified at the level 4 to control for nesting of members and stations within departments. The effects of station-level safety climate across times 1–6 were modeled at level 3. Individual-level emotional exhaustion, depression, and safety compliance were specified at level 2. Time was specified at level 1 capturing repeated measures within individuals. For all hypotheses, station-level safety climate and individual-level emotional exhaustion, both based on the six repeated measures, were introduced as the independent variables along with the time variable. Dependent variables were six repeated measures of individual-level safety compliance behavior (Hypothesis 1), morale (Hypothesis 2), and depression (Hypothesis 3).

Every hypothesis testing model focuses on the interrelations among the independent and dependent variables over time. No particular time effect was hypothesized, but the within-individual-level time variable and its two-way and three-way interactions with safety climate and emotional exhaustion were specified. We aimed to examine whether the hypothesized relationships are consistent or variable over time. For instance, the analysis of the three-way interaction across safety climate, emotional exhaustion, and time enables the examination of whether the interaction between safety climate and emotional exhaustion systematically varies across time. That is, the pattern of how a given dependent variable unfolds over time depending on safety climate and emotional exhaustion can be examined by analyzing the three-way interaction. That being said, it is worth noting that no particular trends of the dependent variables were proposed in the present study. This was because the present study did not entail any intervention effort to alter the conditions that may impact the dependent variables. Although we were open to the possibility that the dependent variables of our interests change in certain directions (i.e., increase or decrease), our main focus was on the interplay between safety climate and emotional exhaustion while the time variable was treated more like a control variable. In the hypothesis testing models, potential variations of the intercept and slope across individuals, stations, and departments were controlled for by specifying random effects across individuals (subscript *i*), stations (subscript *j*), and departments (subscript *k*) for the intercept (i.e., *e*_0ijk_, *r*_00jk_, *u*_00k_) and slope for time (i.e., *e*_1ijk_, *r*_01jk_, *u*_01k_). Also, gender and tenure were included as control variables at the individual-level.

In addition, our multilevel analyses also helped to minimize the potential effects of common method bias. Multilevel analyses account for both within- and between-individual variation in the study variables by examining the hypothesized relationships (i.e., fixed effects) after parsing out the uniqueness of individual trajectories of the repeatedly measured variables and the unique patterns of the hypothesized relationships across individuals and their groups (i.e., random effects). Also, the time-varying conditions that might be systematically confounded with the transient states of first responders were statistically adjusted in our hypothesis tests by controlling for the effects of time.

## Results

Descriptive statistics such as means and standard deviations, and zero-order correlations among the study variables across times 1 through 6 are presented in Table 1. We first conducted a series of multilevel confirmatory factor analyses (MCFAs) at each of the six time points in our study. Items were loaded only on their respective construct and no cross-loading or correlations among the error terms were estimated. As displayed in Table 2, overall, the MCFA results showed acceptable to satisfactory fit (Hu & Bentler, 1999; MacCallum et al., 1996; Mathieu & Taylor, 2006). At each time point, emotional exhaustion and depression were only moderately correlated (*r* = 0.41–0.51). To address concerns regarding the overlap among the emotional exhaustion and depression constructs (Bianchi et al., 2015), we compared the fit of the hypothesized five-factor measurement model to an alternative four-factor model combining the emotional exhaustion and depression items to form a single latent construct. The results indicate a notable deterioration in fit for the four-factor model compared to the hypothesized measurement model (Cheung & Rensvold, 2002; Shaffer et al., 2016). All *χ*^2^ differences were statistically significant (*p* < 0.01) and CFI difference values were all greater than 0.02. These findings support our treatment of emotional exhaustion and depression as separate constructs. The results of the multilevel modeling for repeated measures are presented in Tables 3, 4, and 5.

**Table 1:** Means, standard deviations, and zero-order correlations

	mean (SD)	1	2	3	4	5	6	7	8	9	10	11	12	13	14	15
1. Safety climate T1	4.04 (.74)	.90														
2. Emotional exhaustion T1	2.59 (.88)	− .20	.92													
3. Safety compliance T1	4.50 (.49)	.19	− .23	.62												
4. Morale T1	3.72 (.87)	.60	− .33	.23	.82											
5. Depression T1	1.66 (.77)	− .28	.25	− .12	− .20	.79										
6. Safety climate T2	4.05 (.76)	.62	− .06	.17	.34	− .21	.91									
7. Emotional exhaustion T2	2.51 (.92)	− .09	.52	− .01	− .17	.35	− .13	.94								
8. Safety compliance T2	4.47 (.48)	.11	− .14	.36	.04	− .23	.22	− .19	.66							
9. Morale T2	3.69 (.85)	.33	− .28	.19	.66	− .22	.39	− .37	.19	.81						
10. Depression T2	1.49 (.67)	− .33	.30	− .12	− .33	.42	− .34	.43	− .11	− .38	.89					
11. Safety climate T3	4.06 (.80)	.46	− .11	.08	.27	− .18	.48	− .11	.16	.37	− .26	.92				
12. Emotional exhaustion T3	2.59 (.92)	− .04	.51	− .09	− .17	.29	− .06	.58	− .26	− .25	.26	− .11	.95			
13. Safety compliance T3	4.45 (.51)	.08	− .24	.38	.20	− .28	.04	− .16	.40	.17	− .11	.11	− .19	.74		
14. Morale T3	3.61 (.85)	.26	− .18	.10	.59	− .11	.17	− .09	.06	.63	− .18	.47	− .24	.23	.81	
15. Depression T3	1.61 (.73)	− .20	.19	.01	− .17	.35	− .16	.14	− .20	− .13	.28	− .37	.30	− .09	− .29	.86
16. Safety climate T4	4.01 (.75)	.60	− .15	.12	.36	− .19	.57	− .18	.17	.31	− .22	.42	− .07	.20	.23	− .17
17. Emotional exhaustion T4	2.69 (.87)	− .09	.53	− .06	− .25	.24	− .09	.53	− .12	− .30	.32	− .06	.74	− .19	− .21	.16
18. Safety compliance T4	4.39 (.55)	.15	− .11	.30	.20	− .10	.10	− .11	.31	.18	− .09	.14	− .12	.54	.18	− .07
19. Morale T4	3.59 (.80)	.33	− .32	.22	.63	− .13	.39	− .27	.13	.77	− .26	.38	− .21	.24	.63	− .08
20. Depression T4	1.56 (.75)	− .20	.32	− .19	− .23	.37	− .21	.42	− .13	− .42	.51	− .20	.34	− .19	− .17	.37
21. Safety climate T5	3.97 (.80)	.40	− .01	.01	.20	− .11	.49	− .04	.02	.23	− .20	.39	− .04	.02	.19	.00
22. Emotional exhaustion T5	2.64 (.91)	− .02	.59	− .07	− .15	.20	.00	.48	− .18	− .20	.25	.04	.65	− .22	− .09	.08
23. Safety compliance T5	4.36 (.66)	.15	− .13	.33	.08	− .06	.07	− .08	.32	.08	− .09	.10	− .11	.41	.11	− .05
24. Morale T5	3.49 (.82)	.26	− .32	.22	.51	− .07	.29	− .17	.10	.56	− .25	.22	− .15	.25	.49	− .04
25. Depression T5	1.55 (.73)	− .25	.28	− .08	− .31	.41	− .28	.42	− .21	− .43	.54	− .17	.30	− .24	− .24	.20
26. Safety climate T6	4.06 (.78)	.33	− .14	.11	.30	− .23	.45	− .12	.15	.24	− .25	.35	− .09	.24	.25	− .08
27. Emotional exhaustion T6	2.88 (1.09)	.06	.32	.01	− .02	.12	.07	.23	− .04	− .05	.07	.04	.31	− .03	.03	.07
28. Safety compliance T6	4.28 (.70)	.01	− .11	.26	.03	− .15	.09	− .14	.30	.12	− .09	.06	− .24	.45	.08	.03
29. Morale T6	3.40 (1.03)	.11	− .14	− .05	.24	− .12	.09	− .15	.06	.32	− .09	.21	− .15	.02	.36	− .14
30. Depression T6	1.81 (.97)	.05	.06	− .08	− .07	.19	− .12	.17	− .07	− .13	.30	.04	.12	− .09	.03	.10

	16	17	18	19	20	21	22	23	24	25	26	27	28	29	30
16. Safety climate T4	.93														
17. Emotional exhaustion T4	− .19	.95													
18. Safety compliance T4	.35	− .18	.73												
19. Morale T4	.47	− .34	.26	.80											
20. Depression T4	− .26	.34	− .24	− .32	.87										
21. Safety climate T5	.51	− .10	.16	.33	− .12	.93									
22. Emotional exhaustion T5	− .03	.74	− .04	− .17	.23	.03	.94								
23. Safety compliance T5	.22	− .14	.51	.17	− .20	.29	− .08	.83							
24. Morale T5	.34	− .29	.23	.67	− .31	.32	− .27	.40	.83						
25. Depression T5	− .27	.26	− .23	− .35	.66	− .20	.27	− .22	− .29	.84					
26. Safety climate T6	.44	− .12	.17	.27	− .19	.45	− .06	.23	.32	− .29	.91				
27. Emotional exhaustion T6	.01	.47	− .02	− .06	.07	.02	.56	− .05	− .16	.11	.18	.94			
28. Safety compliance T6	.14	− .22	.40	.13	− .30	.15	− .12	.56	.14	− .21	.28	− .03	.80		
29. Morale T6	.20	− .24	.00	.40	− .16	.20	− .18	.11	.35	− .16	.04	− .53	.07	.84	
30. Depression T6	− .07	.20	− .04	− .09	.28	− .17	.21	− .29	− .30	.37	− .23	.38	− .30	− .25	.94

Means, standard deviations, and zero-order correlations

*r*s equal or greater than .18 are statistically significant at *p* < .01, *r*s smaller than .18 and equal or greater than .14 are statistically significant at *p* < .05, Values on a diagonal are Cronbach’s αs

**Table 2:** Multi-level confirmatory factor analyses (MCFAs) for measurement models

	Time 1	Time 2	Time 3	Time 4	Time 5	Time 6
A. Models based on five constructs: safety climate, emotional exhaustion, safety compliance, morale, and depression						
*χ*^2^	1024.10	744.80	868.44	889.31	936.85	728.84
*df*	381	381	381	381	381	381
CFI	.874	.911	.894	.905	.885	.902
RMSEA	.051	.037	.043	.043	.045	.036
SRMR _within_	.056	.059	.063	.057	.061	.064
SRMR _between_	.085	.101	.062	.038	.048	.058
*r*_(emotional exhaustion and depression)_	.50	.51	.47	.46	.44	.41
B. Models in which emotional exhaustion and depression were merged						
*χ*^2^	1208.38	992.17	1008.68	1105.70	1148.98	957.13
*df*	385	385	385	385	385	385
CFI	.840	.851	.864	.865	.843	.839
RMSEA	.057	.047	.048	.051	.053	.046
SRMR _within_	.065	.068	.075	.068	.083	.085
SRMR _between_	.085	.101	.062	.038	.048	.058
*A–B Model comparison*						
Δ*χ*^2^	184.28	247.37	140.24	216.39	212.13	228.29
Δ CFI	.03	.06	.03	.04	.04	.06

All Δ*χ*^2^ were statistically significant (*p* < .01) and all Δ CFI values were greater than .02 (Cheung & Rensvold, 2002), suggesting significant model fit deterioration after the merge of the emotional exhaustion and depression constructs

**Table 3:** Repeated measure multilevel modeling results: DV = safety compliance

		Coefficients (standard error)	95% confidence interval	*df*	*p*- value
*b*_*0*_	Intercept	4.98 (.14)^**^	4.71–5.25	19.26	< .01
*b*_*1*_	Time	− .08 (.03)^**^	− .14 to − .03	20.74	.01
*b*_*2*_	Safety climate	.27 (.12)^*^	.03 to .52	322.70	.03
*b*_*3*_	Emotional exhaustion	− .12 (.03)^**^	− .18 to − .05	551.50	.00
*b*_*4*_	Time × safety climate	.03 (.02)	− .01 to .07	176.30	.13
*b*_*5*_	Time × emotional exhaustion	.02 (.01)^*^	.00 – .03	207.70	.04
*b*_*6*_	Safety climate × emotional exhaustion	.02 (.01)^**^	− .17 to − .03	143.00	.01
*b*_*7*_	Gender	− .06 (.08)	− .21 to .09	653.50	.44
*b*_*8*_	Tenure	− .01 (.00)^*^	− .02 to .00	1119.00	.01
Random effects variance (standard deviation)					
*e*_0ijk_	Individual-level random intercept = .06 (.25)				
*e*_1ijk_	Individual-level random slope for time = .01 (.09)				
*r*_00jk_	Station-level random intercept = .01 (.12)				
*r*_01jk_	Station-level random intercept = .00 (.01)				
*u*_00k_	Department-level random intercept = .01 (.12)				
*u*_01k_	Department-level random intercept = .00 (.02)				
Residual variance (standard deviation) = .16 (.41)					

^**^ *p* < .01, ^*^ *p* < .05, degrees of freedom and statistical significance testing were based on Satterthwaite’s approximation method (Hrong-Tai Fai and Cornelius, 1996; Kuznetsova et al., 2017; Satterthwaite, 1946)

**Table 4:** Repeated measure multilevel modeling results: DV = morale

		Coefficients (standard error)	95% confidence interval	*df*	*p*-value
*b*_*0*_	Intercept	4.05 (.22)^**^	3.63–4.47	15.95	.00
*b*_*1*_	Time	.09 (.03)^**^	.03–.16	463.30	.00
*b*_*2*_	Safety climate	.35 (.17)^*^	.02–.68	375.80	.04
*b*_*3*_	Emotional exhaustion	− .14 (.05)^*^	− .23 to − .04	757.80	.01
*b*_*4*_	Time × safety climate	− .04 (.03)	− .10 to .01	168.20	.12
*b*_*5*_	Time × emotional exhaustion	− .05 (.01)^**^	− .07 to − .03	688.60	.00
*b*_*6*_	Safety climate × emotional exhaustion	.03 (.05)	− .07 to .12	1216.00	.58
*b*_*7*_	Gender	.15 (.12)	− .08 to .40	201.60	.22
*b*_*8*_	Tenure	− .01 (.01)	− .03 to .00	169.40	.06
Random effects variance (standard deviation)					
*e*_0ijk_	Individual-level random intercept = .43 (.65)				
*e*_1ijk_	Individual-level random slope for time = .01 (.12)				
*r*_00jk_	Station-level random intercept = .03 (.17)				
*r*_01jk_	Station-level random intercept = .00 (.02)				
*u*_00k_	Department-level random intercept = .04 (.20)				
*u*_01k_	Department-level random intercept = .00 (.00)				
Residual variance (standard deviation) = .27 (.52)					

^**^ *p* < .01, ^*^ *p* < .05, degrees of freedom and statistical significance testing were based on Satterthwaite’s approximation method (Hrong-Tai Fai and Cornelius, 1996; Kuznetsova et al., 2017; Satterthwaite, 1946)

**Table 5:** Repeated measure multilevel modeling results: DV = depression

		Coefficients (standard error)	95% confidence interval	*df*	*p*-value
*b*_*0*_	Intercept	1.34 (.24)^**^	.90–1.75	5.11	.00
*b*_*1*_	Time	− .10 (.04)^*^	− .17 to − .02	8.24	.04
*b*_*2*_	Safety climate	.39 (.30)	− .18 to .97	541.95	.19
*b*_*3*_	Emotional exhaustion	.11 (.05)^*^	.01–.21	543.48	.03
*b*_*4*_	Time × safety climate	− .18 (.08)^*^	− .34 to − .03	619.26	.02
*b*_*5*_	Time × emotional exhaustion	.04 (.01)^**^	.01–.06	602.57	.00
*b*_*6*_	Safety climate × emotional exhaustion	− .22 (.10)^*^	− .42 to .02	677.21	.03
*b*_*7*_	Time × safety climate × emotional exhaustion	.06 (.03)^*^	.01–.11	844.98	.02
*b*_*8*_	Gender	.03 (.10)	− .17 to.21	200.95	.76
*b*_*9*_	Tenure	.00 (.01)	− .01 to .01	172.87	.52
Random effects variance (standard deviation)					
*e*_0ijk_	Individual-level random intercept = .15 (.39)				
*e*_1ijk_	Individual-level random slope for time = .01 (.09)				
*r*_00jk_	Station-level random intercept = .00 (.05)				
*r*_01jk_	Station-level random intercept = .00 (.03)				
*u*_00k_	Department-level random intercept = .09 (.30)				
*u*_01k_	Department-level random intercept = .00 (.04)				
Residual Variance (Standard Deviation) = .35 (.59)					

^**^ *p* < .01, ^*^ *p* < .05, degrees of freedom and statistical significance testing were based on Satterthwaite’s approximation method (Hrong-Tai Fai and Cornelius, 1996; Kuznetsova et al., 2017; Satterthwaite, 1946)

**Fig. 3:**
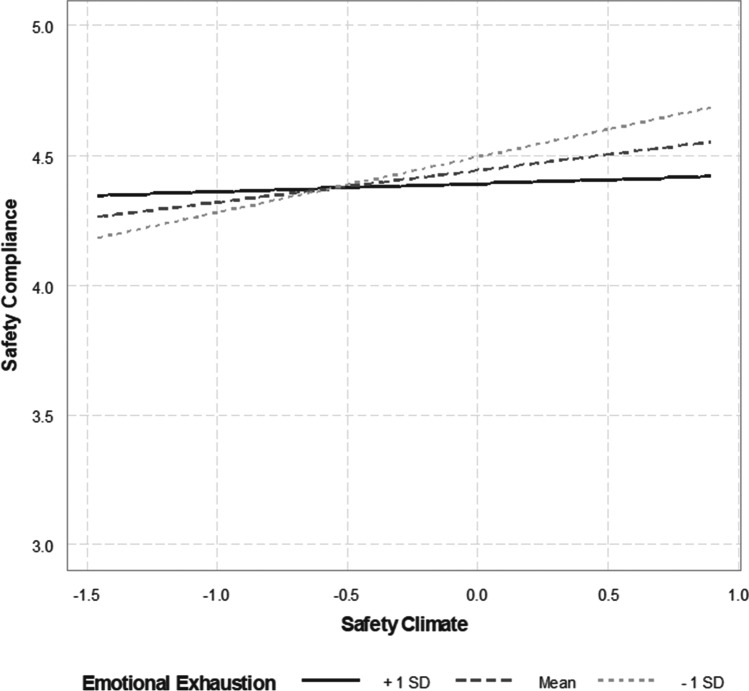
The relationship between safety climate and safety compliance

In testing Hypothesis 1, we found that the three-way interaction among safety climate, emotional exhaustion, and time was not significant in relation to safety compliance behaviors (coefficient =  − 0.03, SE = 0.02, *p* = 0.14). Thus, a simplified model without the three-way interaction term was specified and the results are presented in Table 3. Hypothesis 1 primarily addresses the moderating effect of emotional exhaustion on the relationship between safety climate and safety compliance behaviors that is represented by the two-way interaction between safety climate and emotional exhaustion. The interaction was statistically significant (*b*_*6—*DV*:* safety compliance_ = 0.02, SE = 0.01, *p* < 0.01; Table 3) such that higher safety climate was associated with greater safety compliance behaviors regardless of time, while this effect was attenuated when emotional exhaustion was higher (Fig. 3). Therefore, Hypothesis 1 was supported.

**Fig. 4:**
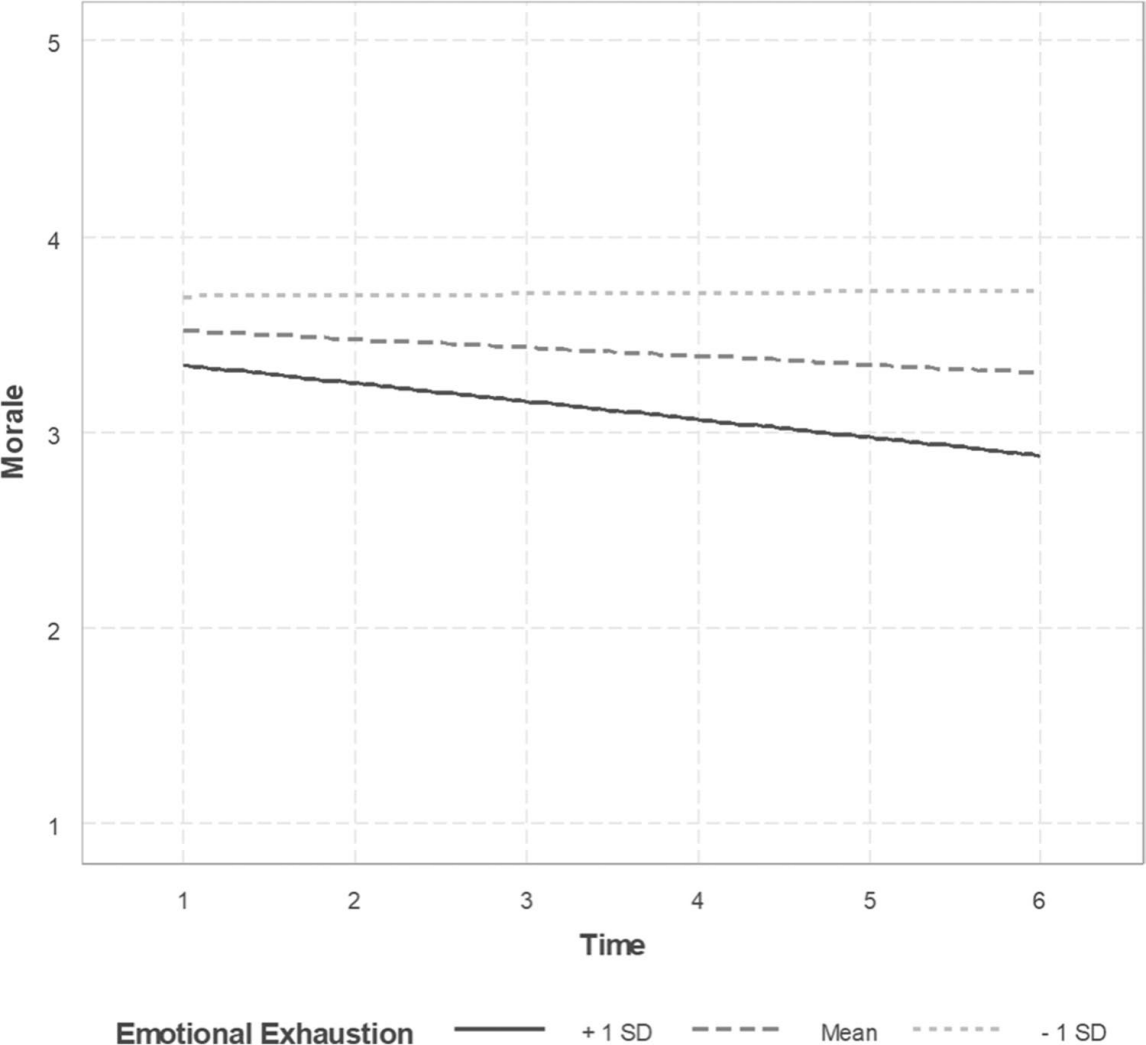
The trend of morale across 6 months depending on the level of emotional exhaustion

In testing Hypothesis 2, the three-way interaction between safety climate, emotional exhaustion, and time was also not significant in relation to morale (coefficient =  − 0.03, SE = 0.02, *p* = 0.30). Thus, a simplified model without the three-way interaction term was specified and the results are presented in Table 4. Hypothesis 2 primarily addresses the moderating effect of safety climate on the relationship between emotional exhaustion and morale, which is represented by the two-way interaction between safety climate and emotional exhaustion. The interaction was not statistically significant (*b*_*6—*DV: morale_ = 0.03, SE = 0.05, *p* = 0.58; Table 4) and Hypothesis 2 was not supported. That being said, the two-way interaction between time and emotional exhaustion was statistically significant (*b*_*5—*DV: morale_ = -0.05, SE = 0.01, *p* < 0.01; Table 4) such that morale tended to decline over time when emotional exhaustion was higher while morale tended to be stable over time when emotional exhaustion was lower. Also, higher emotional exhaustion was consistently associated with a lower absolute level of morale in general. These findings are depicted in Fig. 4. It is also worth noting that safety climate had a significant and positive main effect on morale (*b*_*2*—DV: morale_ = 0.35, SE = 0.17, *p* < 0.05; Table 4). Although Hypothesis 2 was not supported, these findings indicate a detrimental effect of emotional exhaustion on morale over time and a protective effect of safety climate on morale regardless of time.

**Fig. 5:**
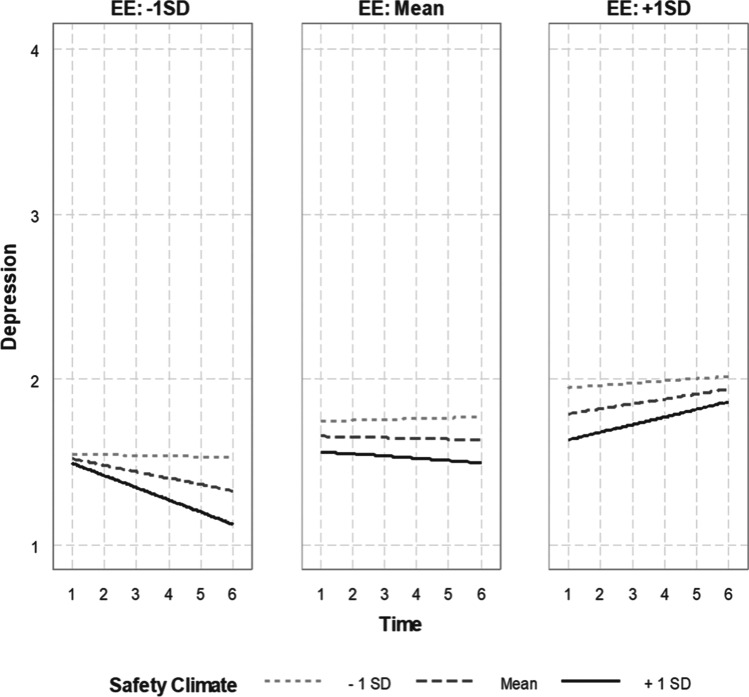
The interaction effects of emotional exhaustion and safety climate on EMS first responder’s depression across 6 months*.* EE, emotional exhaustion

In testing Hypothesis 3, the three-way interaction between safety climate, emotional exhaustion, and time was statistically significant in relation to depression (*b*_*7—*DV: depression_ = 0.06, SE = 0.03, *p* < 0.05; Table 5). When emotional exhaustion was low, high safety climate was associated with decreasing depression over time. When emotional exhaustion was at the average, depression tended to be stable over time, while higher safety climate was associated with lower absolute level of depression in general. When emotional exhaustion was high, an increasing trend of depression was found that was further enhanced as safety climate increased. However, the absolute level of depression was consistently lower when safety climate was higher. These findings, visually depicted in Fig. 5, suggest that emotional exhaustion of EMS first responders can put them in a higher risk of increasing depression, while safety climate can buffer the exacerbation of depression over time. However, the protective effect of safety climate may be obscured when EMS first responders experience intense emotional exhaustion. Jointly considered, Hypothesis 3 is conditionally supported.

As a robustness check, we re-tested the hypotheses removing the gender and tenure control variables. The results without controls were nearly identical in terms of the magnitude, direction, and statistical significance ruling out the possibility that the results can be attributed to the inclusion of the control variables (Spector & Brannick, 2011). As a second robustness check, we used list-wise deletion to restrict our sample to complete cases and conducted the analyses using only those complete cases. The pattern of findings for all hypothesis tests was similar in magnitude, direction, and statistical significance suggesting that our imputation with PMM to address missing data was not likely to affect the results.

## Discussion

Various job demands challenge the safety and wellbeing of EMS first responders. Studies suggest that an adverse event like the COVID-19 pandemic can necessitate enhanced safety regulations and stringent compliance to safety protocols for first responders, further intensifying their job demands (Coto et al., 2020; Ventura et al., 2020). When these situational demands are coupled with sufficient organizational resources and effective safety leadership, safety climate may be bolstered. Also, each station may respond and react differently to safety, health, and wellbeing-related issues. Some, but not all, stations may prioritize safety and health of the public over those of first responders and therefore have more first responders who are vulnerable to safety and health risks than those in other stations. Moreover, the interpretation and experience of adverse working conditions may differ across individuals. The present study focused on safety climate as an organizational context and emotional exhaustion as a psychological context, and examined how they jointly affect EMS first responders’ safety behaviors and wellbeing.

Our findings showed that the overall positive relationship between safety climate and safety compliance behaviors can be attenuated when EMS first responders experience high emotional exhaustion. This suggests that actively managing the psychological context of individual workers is important to optimize the effect of safety climate. Also, the findings indicate that safety climate did not moderate the relationship between emotional exhaustion and morale. Instead, emotional exhaustion was negatively associated with morale while safety climate was positively associated with morale. The findings further indicate that EMS first responders experienced exacerbated depression when their emotional exhaustion levels were high. Safety climate was associated with decreased depression when emotional exhaustion was within a low to medium range, along with lower absolute levels of depression across the entire range of emotional exhaustion. These findings suggest that promotion of safety climate and mitigation of emotional exhaustion can augment EMS first responders’ wellbeing across both work and personal domains.

### Theoretical Implications

#### Job Demands–Resources (JD-R) Theory

The present study tests and extends tenets of the JD-R (Demerouti & Bakker, 2011; Demerouti et al., 2001) in two ways. First, the results for Hypothesis 1 provide an opposing piece of evidence to the JD-R’s coping hypothesis (Bakker et al., 2010; Hakanen et al., 2005) which contends that high job demands can amplify the saliency of job resources, subsequently strengthening the relationship between job resources and positive outcomes like motivation and engagement. Instead, the results are congruent with the findings of previous studies (Bakker & Costa, 2014; Leitão et al., 2021; Liang, 2012) which showed that the relationship between job resources and positive outcomes can be compromised when job demands are high. The findings suggest that the moderating effect of job demands on the relationship between job resources and outcomes is not unidirectional. Also, when job demands undermine individuals’ ability to make the best use of job resources, the impact of job resources can be reduced.

Second, the findings provide conditional support for the buffer hypothesis of JD-R theory, which contends that the detrimental relationship between job demands and strain can be neutralized or negated by job resources (Bakker et al., 2003, 2005; Xanthopoulou et al., 2007). The results for Hypothesis 3 are consistent with the buffer hypothesis. Safety climate in the form of support from supervisors for overall occupational health and wellbeing can buffer employees from the detrimental effects of emotional exhaustion and depression. Indeed, employees are likely to experience positive emotions when they perceive that they are genuinely supported and protected by their supervisors (Huang et al., 2016). This provides an important form of psychological support that helps them to better address the strain from their work.

Nevertheless, it is worth noting that when emotional exhaustion is substantially high, depression may be exacerbated over time even when safety climate is high. Organizational climate reflects the norms, expectations, and perceptions that are shared among employees and promote collective behaviors in accordance with their organizational policies and practices. Said differently, it may be perceived as a demand in certain conditions (e.g., Katz-Navon et al., 2020). Specifically, in response to the demands of an organization with a high safety climate, employees are likely to expend resources such as complying with all safety rules and regulations and exercising extra caution in hazardous work environments. When they are experiencing emotional exhaustion, employees may suffer from a substantial loss of resources, making it harder to adhere to the safety climate–induced expectations. This reasoning underscores the importance of simultaneously examining both the organizational context (safety climate) and psychological context (emotional exhaustion) jointly.

Moreover, the results for Hypothesis 2 did not detect a significant buffering effect of safety climate on the relationship between emotional exhaustion and morale. Presumably, there might be job resources that are more salient to EMS first responders’ wellbeing in terms of morale and depression. For example, psychosocial safety climate (Dollard & Bakker, 2010; Dollard & McTernan, 2011; Zadow & Dollard, 2016), emphasizing members’ psychological health and safety rather than physical and industrial safety, might have a stronger mitigating effect. The buffering effect of safety climate on the relationship between emotional exhaustion and depression, that is partially supported, may be due to a more general level of support from station supervisors than the station’s safety climate. For example, social support and fair treatment of EMS first responders, and responsible and caring attitudes of supervisors may influence how employees responded to the questions regarding the station’s safety climate. Unfortunately, the data do not enable us to further tease apart the unique effects of safety climate from a more general climate for social support or supervisor support. These unique effects should be examined in future studies.

#### Safety Climate

Additionally, the present study advances the safety climate literature in two ways. First, it extends beyond past studies regarding the impact of safety climate (e.g. Huang et al., 2016; Nielsen et al., 2011; Taylor et al., 2019) by showing the potential protective main effect of safety climate on EMS first responders’ safety compliance behavior and morale. It also shows the potential buffering effect of safety climate on the relationship between EMS worker emotional exhaustion and depression. The results corroborate the view that the impact of safety climate can go above and beyond workplace safety outcomes (Huang et al., 2016).

Second, the present study incorporated individual workers’ psychological context (i.e., emotional exhaustion) as a potential boundary condition for safety climate. Our findings indicate that EMS first responders’ capacity to respond to their station’s safety climate and engage in safety compliance behaviors can systematically vary depending on their own psychological context in terms of the emotional exhaustion they experience from work. This provides some explanation for why individual safety behaviors and safety outcomes vary within a station even though members are exposed to the same safety climate expectations.

Additionally, the present study contributes to elaborating the burnout model (Maslach & Leiter, 2016) by confirming that emotional exhaustion, a key dimension of burnout, is not an ultimate end-outcome of occupational stress due to various job demands. Burnout may have an aggravating effect over time on workers’ wellbeing at work (e.g., morale) and wellbeing in personal/global domains (e.g., depression). Our study showed how emotional exhaustion can be associated with morale and how depression may unfold over time in conjunction with safety climate and emotional exhaustion. It is suggested that emotional exhaustion can indeed have enduring spillover effects if left unattended.

### Practical Implications

To promote safety climate and its optimal functioning, it is necessary to consider the unique psychological context of employees. Factors like emotional exhaustion may impair members’ capacity to judge risks, and fully engage in practices that protect their own health (Giordano et al., 2021; Leiter & Robichaud, 1997). Meanwhile, emotional exhaustion may affect individuals differently, depending on individual-specific aspects such as efficacy, resilience, and support from family. For the optimal tailoring of organizational safety and wellbeing promotion efforts, it is pivotal to gain a comprehensive understanding of the needs, concerns, preferences, and resources of individual employees. To this end, the integrative and participatory approach that involves individual employees in designing and implementing organizational improvement and change process can be helpful (Davis et al., 2020; Lee et al, 2021, 2019a, b, c).

Also, the findings from the present study corroborate the previous research concerning the importance of supervisory behavior and employee wellbeing (Inceoglu et al., 2018). Supervisors of EMS first responders who actively support safety initiatives within their fire stations play a central role counterbalancing the demands of the work environment, facilitating safety compliance behaviors, and preventing the decline of morale and worsening of depression. The support from senior leadership and key decision-makers to group-level leader (e.g., direct supervisors) can be critical for fostering station safety climate. Organizational interventions to strengthen safety climate can be considered. Example strategies include safety leadership training (Goldenhar et al., 2019), after action reviews (Allen et al., 2010), and supervisor safety communication training (Huang et al., 2018; Zohar & Polachek, 2014).

Plus, we observed the deterioration of morale and exacerbation of depression along with emotional exhaustion over the 6-month study period. This finding underscores the importance of monitoring the behavioral and psychological after-effect of emotional exhaustion among workers with high job demands. Programs to aid EMS first responders’ restoration of physical and psychological resources such as stress coping skill training, time off, professional services, or coaching would be helpful.

## Limitations and Future Directions

The present study has some limitations and opportunities for future research. First, we had a less than optimal recruitment rate (i.e., 26.00% = [final sample size of 208] / [entire roster size of the three participating departments of 800]) and survey completion rate (70.99%) across the six waves of data collection. As noted, the COVID-19 pandemic may have heightened work demands, hindering EMS first responders’ survey participation. We note that caution is needed in generalizing the study findings to the entire EMS first responder population. Second, response patterns could be influenced by fluctuations in mood or overall response styles leading to response bias. This issue is particularly relevant to our study because all the data are based on survey responses. Future studies can consider objective indicators of safety (e.g., incidents, injuries) and wellbeing (e.g., attendance, health outcomes) to ease concerns regarding response bias and extend the study’s findings. Third, Fu et al. (2021) offer an alternative perspective on the strain of the COVID-19 pandemic finding that workers in general (not medical specific) acclimate to the environmental stressors and experience less threats to psychological wellbeing unless there is an acceleration in the intensity of the cases in one’s home state. More needs to be done to investigate the evolution of COVID 19 and preparedness for future public health crises, and how that might impact the onset and development of emotional exhaustion and depression. Fourth, our study focused exclusively on the emotional exhaustion dimension of burnout. The unique roles of the remaining dimensions of burnout—depersonalization and reduced personal accomplishment—should be examined in relation to safety behaviors and wellbeing. Future research could also consider alternative perspectives on safety within organizational contexts (e.g., safety leadership, communication quality, co-worker support) which may also be relevant to understanding the impact of emotional exhaustion.

## Conclusion

The demanding work context of EMS first responders has been shown to compromise their safety, health, and wellbeing. Safety climate is an important organizational resource that may enhance EMS first responders’ safety and wellbeing. However, our findings demonstrate that emotional exhaustion can undermine these protective benefits by weakening compliance with safety protocols. Moreover, our findings suggest that safety climate only lessens the likelihood of emotional exhaustion being associated with depression over time when emotional exhaustion is not severe. Facilitating station-level safety climate and addressing individual EMS first responders’ emotional exhaustion is critical for safeguarding various aspects of occupational safety and health in this extraordinarily demanding work environment.

## Data Availability

Due to privacy and ethical concerns, supporting data cannot be made openly available. Further information about the data and conditions for access are available from the corresponding author.

## Appendix

### Scales and Items

#### Safety Climate—FOCUS supervisor support (Taylor et al., 2019)

Our direct supervisor prioritizes rest and rehabilitation on scene.

My direct supervisor puts a high emphasis on safety training.

I have confidence in my command/company level officers to keep me safe.

On our crew, people expect one another to wear their PPE.

Our house does a good job of carrying out its safety policies.

In our firehouse, we talk about safety on a consistent basis.

My direct supervisor takes my safety concerns seriously.

#### Burnout—Emotional Exhaustion (Maslach & Jackson, 1981)

I feel emotionally drained from this kind of work.

I feel used up at the end of a run.

I feel fatigued when I get up in the morning and have to face another day on the job.

I feel burned out doing this kind of work.

Working with people all day is really a strain for me.

I feel frustrated by my job.

I feel I'm working too hard on my job.

Working with people directly puts too much stress on me.

I feel like I'm at the end of my rope.

#### EMS Safety Compliance (Newly Developed)

I ensure the scene is safe on each call.

I wear PPE (e.g., gloves, eye protection, mask, etc.) while engaging in patient care activities.

I ensure that my PPE is properly discarded and/or cleaned at the end of each call.

I clean the patient care compartment in between calls.

I shower and change my clothes after my shift.

I wear my seatbelt while traveling to and from an event.

I report my exposures to infection control.

#### Morale (Sexton et al., 2006)

I like my job.

Morale is high here.

Working here is like being part of a family.

This department is a good place to work.

#### Depression (Kroenke et al., 2007)

Over the last 2 weeks, how often have you been bothered by the following problems?

Little interest or pleasure in doing things.

Feeling down, depressed, or hopeless.

## Footnotes

1 Although the average Cronbach’s alpha was above .70, Cronbach’s alphas for times 1 and 2 were .62 and .66, respectively. This may be attributable to the fact that individual items indicate a distinct safety compliance behavior and the items may not be interchangeable even though they collectively indicate the same construct. Similar reliabilities have been commonly reported for safety behavior scales (e.g., Huang et al., 2014; Lee et al., 2019a, 2019b, 2019c; Zohar et al., 2015) and were not unacceptably low (*α* < 0.50; George & Mallery, 2003; Kline, 2000).
